# Pentadecanoic Acid (C15:0), an Essential Fatty Acid, Shares Clinically Relevant Cell-Based Activities with Leading Longevity-Enhancing Compounds

**DOI:** 10.3390/nu15214607

**Published:** 2023-10-30

**Authors:** Stephanie Venn-Watson, Nicholas J. Schork

**Affiliations:** 1 Epitracker Inc., San Diego, CA 92106, USA; 2 Seraphina Therapeutics, Inc., San Diego, CA 92106, USA; nschork@tgen.org; 3 Translational Genomics Research Institute (TGen), City of Hope, Phoenix, AZ 85004, USA

**Keywords:** C15:0, pentadecanoic acid, rapamycin, metformin, acarbose, longevity, essential fatty acid

## Abstract

Pentadecanoic acid (C15:0) is an essential odd-chain saturated fatty acid with broad activities relevant to protecting cardiometabolic, immune, and liver health. C15:0 activates AMPK and inhibits mTOR, both of which are core components of the human longevity pathway. To assess the potential for C15:0 to enhance processes associated with longevity and healthspan, we used human cell-based molecular phenotyping assays to compare C15:0 with three longevity-enhancing candidates: acarbose, metformin, and rapamycin. C15:0 (n = 36 activities in 10 of 12 cell systems) and rapamycin (n = 32 activities in 12 of 12 systems) had the most clinically relevant, dose-dependent activities. At their optimal doses, C15:0 (17 µM) and rapamycin (9 µM) shared 24 activities across 10 cell systems, including anti-inflammatory (e.g., lowered MCP-1, TNFα, IL-10, IL-17A/F), antifibrotic, and anticancer activities, which are further supported by previously published in vitro and in vivo studies. Paired with prior demonstrated abilities for C15:0 to target longevity pathways, hallmarks of aging, aging rate biomarkers, and core components of type 2 diabetes, heart disease, cancer, and nonalcoholic fatty liver disease, our results support C15:0 as an essential nutrient with activities equivalent to, or surpassing, leading longevity-enhancing candidate compounds.

## 1. Introduction

Pentadecanoic acid (C15:0), an odd-chain saturated fatty acid, has mounting evidence of being essential to supporting cardiometabolic and liver health [1,2]. People with low circulating C15:0 concentrations have a higher risk of having or developing type 2 diabetes, heart disease, nonalcoholic fatty liver disease, and nonalcoholic steatohepatitis, as well as specific types of cancer [3,4,5,6,7,8,9,10,11,12,13,14,15,16,17,18,19]. C15:0 is present in 1 to 3% of dairy fat, and as a primarily exogenous molecule, circulating C15:0 concentrations are reflective of dietary C15:0 intake [20,21]. Epidemiological, in vivo, and in vitro studies support the notion that 100 to 300 mg of C15:0 is needed daily to effectively achieve and maintain active circulating C15:0 concentrations of 10 to 30 µM [2]. As such, there is building evidence for minimum daily C15:0 requirements and defined C15:0 nutritional deficiencies [2].

Pure C15:0 is a pleiotropic nutrient with broad-reaching mechanisms of action, including AMPK and PPAR-α/δ activation, as well as mTOR, JAK-STAT, and HDAC-6 inhibition [2,22,23,24,25]. These mechanisms are aligned with the demonstrated anti-inflammatory, antifibrotic, and anticancer activities of C15:0 in vitro and in vivo [2,25,26,27,28]. They are also consistent with epidemiological studies linking higher circulating C15:0 concentrations to lower cholesterol, triglycerides, ApoA1 and ApoB, liver enzymes, C-reactive protein, adipokines, body mass index; and improved insulin sensitivity and β cell function in humans [29,30,31,32,33,34].

Beyond targeting key receptors, C15:0 is a stable saturated fat that is readily incorporated into the lipid bilayers of cell membranes, including red blood cells, to lower the risk of lipid peroxidation and premature lysis [2,35,36,37,38]. Further, C15:0 has antimicrobial properties, including growth inhibition of pathogenic bacteria and fungi [39,40]. Combined, these broad positive activities of C15:0 support its role as an essential nutrient to support long-term physiological health.

There is tremendous interest in developing and testing interventions that can simultaneously prevent or mitigate multiple age-related diseases and thereby enhance healthspan and longevity [41,42,43,44]. The belief that such interventions can be developed is rooted in what is often referred to as the “geroscience hypothesis” [45]. This hypothesis argues that there are likely mechanisms that govern the rate at which individuals age, and if one could identify these mechanisms and somehow slow them through an intervention, then many disease processes known to be exacerbated by aging could be mitigated simultaneously [44,45,46,47].

The belief that there are specific mechanisms contributing to aging, the aging rate, age-related diseases, and longevity, that, if modulated, could have healthy aging effects has been strengthened by focused studies exploring various “hallmarks” of aging (such as mitochondrial function and DNA repair capacity), as well as candidate measures of an actual aging rate based on, e.g., epigenetic clocks [48,49,50]. This work has motivated researchers to identify interventions that affect these hallmarks and clocks or impact aging, aging-related diseases generally and longevity, and test them for their effects on clinically relevant disease endpoints [51]. In fact, over the past decade, the development of interventions to address the condition of aging has transitioned from fringe science to FDA-approved clinical trials, in which “aging” is listed as a valid targeted condition for many interventions being tested [52].

Many interventions have been shown to enhance longevity in non-human species [41,53] as well as protect humans from different age-related diseases, or at least mitigate their effects [41]. The mechanisms of action of many of these interventions, however, are often obscure, and whether they truly have health or lifespan-enhancing capabilities in humans is unknown [48]. Among the hundreds of interventions (mostly compounds and dietary manipulations such as caloric restriction) that have been evaluated, several, including rapamycin, metformin, and acarbose, have demonstrated an ability to safely target various hallmarks of aging, manage and treat chronic aging-related conditions, and extend mammalian lifespans [54]. Among these, rapamycin is often considered the most promising compound [55].

Rapamycin helps stabilize cell function by inhibiting the mammalian target of the rapamycin (mTOR) pathway [56]. Rapamycin has been shown to be an effective immunomodulator and antiproliferative compound, resulting in demonstrated anti-inflammatory, antifibrotic, and anticancer activities in vitro and in vivo [57,58,59]. Rapamycin’s combination of pleiotropic activities, linked primarily to its mTOR-inhibiting effect, may explain how this compound has been able to extend lifespan in both female and male mice [60]. Given these encouraging studies, there has been a strong push to evaluate rapamycin as a healthspan and longevity-enhancing intervention in humans [55].

Although metformin has been used as a medicine for nearly four centuries, it has been more recently evaluated as a candidate longevity-enhancing compound [61]. Metformin effectively and safely lowers blood sugar in humans and, as a result, is the most common first-line management of type 2 diabetes globally [62]. Metformin primarily works by activating AMPK (adenosine monophosphate-activated protein kinase), which serves as a reliable sensor and regulator of cellular energy [63]. Beyond lowering blood sugar, metformin targets multiple hallmarks of aging that help to lower inflammation, lower oxidative stress, and repair overall cell function [64]. In pathways known to impact longevity, AMPK activation is upstream of mTOR, giving metformin the potential to have broader anti-aging effects than rapamycin, including as an mTOR inhibitor [57]. While metformin was initially shown to extend lifespan in female and male mice, a more recent meta-analysis concluded that metformin is not significantly associated with overall increases in lifespan in mice [65,66,67]. Metformin, however, is being tested as a longevity-enhancing intervention for humans, including as part of the ongoing Targeting Aging with Metformin (TAME) clinical trial [68].

Similar to metformin, acarbose is used to lower blood sugar in people with type 2 diabetes [69]. While metformin is an AMPK activator that works systemically, acarbose is an α-glucosidase inhibitor that slows the breakdown and digestion of carbohydrates in the gut [70]. By decreasing the absorption of dietary carbohydrates, acarbose effectively lowers postprandial glucose levels, protecting against the numerous negative downstream effects that sustained hyperglycemia has on tissues [71]. Acarbose has been shown to increase the lifespan of mice, especially males, and it has been proposed that the influence of acarbose on gut microbiota may explain its ability to support longer life [72,73,74].

The BioMAP Diversity PLUS system includes a series of independently run and industry-standard pharmacological assays routinely used to screen and compare molecules for activity profiles and clinical indications as well as safety [75]. Specifically, the BioMAP Diversity PLUS system (Eurofins Panlabs, St. Charles, MO, USA) tests molecules across 12 primary human cell-based systems mimicking various disease states and measures the molecule’s effects across 148 clinically relevant biomarkers at four doses. The resulting cell-based phenotypic profile enables valuable insights into potential clinical applications of a compound, as well as identifying shared key activities with other compounds of interest.

As an essential fatty acid, C15:0 should, by definition, support healthspan, and longevity. Further, C15:0 has mTOR-inhibiting and AMPK-activating activities shared with rapamycin and metformin, respectively [22,23]. As such, we compared the primary human cell phenotypic profile of C15:0 with acarbose, metformin, and rapamycin using BioMAP Diversity PLUS, which was conducted at the Eurofins lab, an independent third-party provider of the BioMAP assay, to objectively evaluate common clinically relevant cell-based activities supportive of an expanded healthspan and lifespan. Based on our findings, we then reviewed the literature for further evidence of C15:0 as a longevity-enhancing nutrient.

## 2. Materials and Methods

### 2.1. Basic Eurofins BioMAP Assay System and Cell Exposure Studies

We made use of the Eurofins BioMAP assay system, which has a long history of use in pharmaceutical development, evaluation, and comparisons of newly studied compounds with well-established pharmaceutical compounds [75]. Briefly, the system consists of 148 assays across 12 primary human cell-based systems mimicking various disease states. The Eurofins BioMAP assay is run in an independent Eurofins lab, with users simply providing constructs and compounds used for the screening, reducing potential biases associated with a lab developing or promoting the use of a compound.

Our use of the BioMAP system entailed exposing cells to 4 different concentrations of each of the compounds tested: C15:0 (FA15, 99% pure free fatty acid C15:0 ingredient from Seraphina Therapeutics, Inc.) with concentrations, 1.9, 5.6, 17, 50 µM; rapamycin with concentrations, 0.3, 1, 3, 9 µM; metformin with concentrations, 190, 560, 1700, 5000 µM; and acarbose with concentrations, 1.1, 3.3, 10, 30 µM. C15:0 doses were based on circulating C15:0 concentration ranges from in vivo studies and similar effective concentrations related to PPARα/δ agonist and mitochondrial repair activities [2]. The ascending doses for rapamycin (0.3, 1, 3, 9 µM), metformin (190, 560, 1700, 5000 µM), and acarbose (1.1, 3.3, 10, 30 µM) were based on previously established ranges by Eurofins/DiscoverX (Eurofins Panlabs, St. Charles, MO, USA) as benchmarking compounds in their BioMAP system; specifically, these concentrations provided optimal efficacies without having broad toxicity (defined above, when cytotoxicity was detected in 3 or more of the 12 tested systems).

The BioMAP assay system uses human primary cells at early passages (passage 4 or earlier) to minimize adaptation to cell culture conditions and to preserve physiological signaling responses. All cells were from a pool of multiple donors (n = 2 to 6), commercially purchased, and handled according to the recommendations of the manufacturers. Human blood-derived CD14+ monocytes were differentiated into macrophages in vitro before being added to the lMphg system. Cell types and stimuli used in each system—as well as the overall phenomena they are designed to interrogate—were as follows: 3C system [human umbilical vein endothelial cells (HUVEC) + (IL-1β, TNFα, and IFNγ)] (inflammation); 4H system [HUVEC + (IL-4 and histamine)] (etc.); LPS system [primary blood mononuclear cells (PBMC) and HUVEC + LPS (TLR4 ligand)] (etc.); SAg system [PBMC and HUVEC + TCR ligands] (etc.); BT system [CD19+ B cells and PBMC + (α-IgM and TCR ligands)] (etc.); BF4T system [bronchial epithelial cells and HDFn + (TNFα and IL-4)] (etc.); BE3C system [bronchial epithelial cells + (IL-1β, TNFα, and IFNγ)] (etc.); CASM3C system [coronary artery smooth muscle cells + (IL-1β, TNFα, and IFNγ)] (etc.); HDF3CGF system [HDFn + (IL-1β, TNFα, IFNγ, EGF, bFGF, and PDGF-BB)] (etc.); KF3CT system [keratinocytes and HDFn + (IL-1β, TNFα, IFNγ, and TGFβ)] (etc.); MyoF system [differentiated lung myofibroblasts + (TNFα and TGFβ)] and lMphg system [HUVEC and M1 macrophages + Zymosan (TLR2 ligand)] (etc.).

The assays were based on either single-cell types or co-culture systems. Adherent cell types were cultured in 96- or 384-well plates until confluence, followed by the addition of PBMC (SAg and LPS systems). The BT system consisted of CD19+ B cells co-cultured with PBMC and stimulated with a BCR activator and low levels of TCR stimulation. Test agents prepared in either DMSO (small molecules; final concentration ≤ 0.1%) or PBS (biologics) were added at the indicated concentrations 1 h before stimulation and remained in culture for 24 h or as otherwise indicated (48 h, MyoF system; 72 h, BT system (soluble readouts); 168 h, BT system (secreted IgG)). Each plate contained vehicle controls (e.g., 0.1% DMSO) appropriate for each system.

Direct ELISA was used to measure biomarker levels of cell-associated and cell membrane targets via absorbance. Soluble factors from supernatants were quantified using either HTRF^®^ detection (Eurofins Panlabs, St. Charles, MO, USA), bead-based multiplex immunoassay, or capture ELISA. Overt adverse effects of test agents on cell proliferation and viability (i.e., cytotoxicity) were detected by sulforhodamine B (SRB) staining, for adherent cells, and alamarBlue^®^ (Eurofins Panlabs, St. Charles, MO, USA) reduction for cells in suspension. For proliferation assays, individual cell types were cultured at subconfluence and measured at time points optimized for each system (48 h: 3C and CASM3C systems; 72 h: BT and HDF3CGF systems; 96 h: SAg system). Cytotoxicity for adherent cells was measured by SRB (24 h: 3C, 4H, LPS, SAg, BF4T, BE3C, CASM3C, HDF3CGF, KF3CT, and lMphg systems; 48 h: MyoF system), and by alamarBlue staining for cells in suspension (24 h: SAg system; 42 h: BT system) at the time points indicated.

### 2.2. Determining Significant Biomarker Hits

Quantitative readouts of biomarkers in treated systems were considered “hits” if a biomarker readout was outside the significance envelope. The significance envelope was defined as symmetrical upper and negative lower bound values of log_10_ transformed historical vehicle controls at a 95% confidence interval. Specific biomarker hits are provided in the Supplementary Materials as blue values (S1 Profile Data).

### 2.3. Activity Profile Analysis for Annotated, Dose-Dependent Activities

Biomarker activities were annotated when two or more consecutive concentrations changed in the same direction relative to vehicle controls, were outside of the significance envelope defined by legacy and accepted use of the BioMAP platform and had at least one concentration with an effect size >20% (|log10 ratio| > 0.1). Biomarker key activities were described as modulated if these activities increased in some systems but decreased in others. Cytotoxic conditions were noted when total protein levels decreased by more than 50% (log10 ratio of SRB or alamarBlue levels <−0.3) and were indicated by a thin black arrow above the X-axis. A compound was considered to have broad cytotoxicity when cytotoxicity was detected in three or more systems. Concentrations of test agents with detectable broad cytotoxicity were excluded from biomarker activity annotation and downstream benchmarking, similarity search, and cluster analysis. Antiproliferative effects were defined by an SRB or alamarBlue log10 ratio value <−0.1 from cells plated at a lower density and were indicated by grey arrows above the X-axis. Cytotoxicity and antiproliferative arrows only required one concentration to meet the indicated threshold for profile annotation.

### 2.4. Determining Optimal Concentrations

The optimal concentrations for each of the evaluated compounds were defined as which of the four tested concentrations had the highest number of clinically relevant biomarker hits. Biomarker hits were defined by a measurement outside of the significance envelope and an effect size > 20% (|log10 ratio| > 0.1).

## 3. Results

**C15:0 and rapamycin have the most dose-dependent and clinically relevant activities.** Full cell-based phenotypic BioMAP Diversity PLUS profiles for each of the four compounds are provided in the Supplementary Materials (Table S1). Of the four compounds tested, pure C15:0 (FA15) had the most dose-dependent annotated activities (i.e., had 2 or more consecutive concentrations changed in the same direction relative to vehicle controls, were outside the significance envelope, and had at least one concentration with an effect size >20% (|log10 ratio| > 0.1)) (n = 36), closely followed by rapamycin (n = 32) (Table 1). Metformin had 17 annotated activities, and acarbose had five. As an added reference from a prior study, omega-3 fatty acid eicosapentaenoic acid (EPA) had seven annotated activities [25]. Rapamycin had positive effects across all 12 cell systems, while C15:0 positively affected 10 out of 12 (83%) cell systems. All four compounds (C15:0, rapamycin, metformin, and acarbose) were found to be non-cytotoxic at all concentrations (Supplementary Materials, Table S2).

C15:0 (1.9–50 µM) shared 12 annotated, dose-dependent cell-based activities with rapamycin (0.3–9 µM) across 7 (58%) out of 12 cell systems (Table 2). Specifically, both C15:0 and rapamycin had significant, dose-dependent effects on lowering: HLA-DR and cell proliferation in the 3C system relevant to cardiovascular disease and chronic inflammation; CD38, CD40, and T cell proliferation in the SAg system relevant to autoimmune disease and chronic inflammation; secreted IgG and IL-17F in the BT system relevant to asthma, allergy, cancer, and autoimmunity; tPA in the BE3C system relevant to lung inflammation and COPD; HLA-DR and cell proliferation in the CASM3C system relevant to cardiovascular inflammation and restenosis; PAI-I and fibrotic cell proliferation in the HDF3CGF system relevant to fibrosis and chronic inflammation; VCAM-1 in the MyoF system relevant to fibrosis, chronic inflammation, wound healing, and matrix remodeling.

In contrast, C15:0 (1.9–50 µM) shared only four annotated cell-based activities with metformin (190–5000 µM) across 2 (17%) out of 12 cell systems (Table 2). Specifically, both C15:0 and metformin had significant, dose-dependent effects on lowering: HLA-DR in the 3C system relevant to cardiovascular disease and chronic inflammation; and CD38, CD69, and T cell proliferation in the SAg cell system relevant to autoimmune disease and chronic inflammation. There was one shared dose-dependent activity between C15:0 (1.9–50 µM) and acarbose (1.1–30 µM): lowered fibrotic cell proliferation in the HDF3CGF system relevant to fibrosis and chronic inflammation.

**At their optimal doses, C15:0 shared numerous clinically relevant activities with rapamycin and metformin.** For each compound, we defined the optimal dose as the dose with the highest number of biomarker “hits” (Table 3, Supplementary Table S3). Optimal doses were 17 µM for C15:0 (81 hits), 9 µM for rapamycin (75 hits), 5000 µM for metformin (53 hits), and 30 µM for acarbose (28 hits). Because acarbose had substantially fewer biomarker hits, we limited comparisons of cell-based activities at optimal doses to C15:0 (17 µM), rapamycin (9 µM), and metformin (5000 µM).

At their optimal doses, C15:0 (17 µM) shared 24 significant cell-based activities with rapamycin (9 µM) across 10 (83%) out of 12 cell systems (Table 4). Specifically, both C15:0 and rapamycin significantly lowered: MCP-1, HLA-DR, SRB, and cell proliferation in the 3C system relevant to cardiovascular disease and chronic inflammation; MCP-1, VCAM-1, CD40, tissue factor and SRB in the LPS system relevant to cardiovascular disease and chronic inflammation; CD38, CD40, T cell proliferation, and SRB in the SAg system relevant to autoimmune disease and chronic inflammation; secreted IgG, IL-17A, IL-17F, and TNFα in the BT system relevant to asthma, allergy, cancer, and autoimmunity; PAI-I in the BE3C system relevant to lung inflammation and COPD; HLA-DR and cell proliferation in the CASM3C system relevant to cardiovascular inflammation and restenosis; PAI-I and fibrotic cell proliferation in the HDF3CGF system relevant to fibrosis and chronic inflammation; PAI-I in the KF3CT system relevant to psoriasis and dermatitis; VCAM-1 in the MyoF system relevant to fibrosis, chronic inflammation, wound healing and matrix remodeling; and IL-10 in the/Mphg system relevant to cardiovascular inflammation, restenosis, and chronic inflammation.

At their optimal doses, C15:0 (17 µM) shared 11 significant cell-based activities with metformin (5000 µM) across 5 (42%) out of 12 cell systems (Table 4). Specifically, both C15:0 and metformin significantly lowered: HLA-DR in the 3C system relevant to cardiovascular disease and chronic inflammation; MCP-1, CD40, and CD69 in the LPS cell system relevant to cardiovascular disease and chronic inflammation; CD38, CD40, CD69, and T cell proliferation in the SAg cell system relevant to autoimmune disease and chronic inflammation; IgG and TNFα in the BT system relevant to asthma, allergy, cancer, and autoimmunity; and VCAM-1 in the HDF3CGF system relevant to fibrosis and chronic inflammation.

## 4. Discussion

Using the BioMAP Diversity PLUS system, our study demonstrates numerous clinically relevant and cell-based activities that are shared between C15:0, an essential dietary fat, and rapamycin, a leading longevity-enhancing compound. Our study’s findings are particularly remarkable given that C15:0 is a common fatty acid naturally present in milk fat, while rapamycin is a rare compound discovered from bacteria growing on Easter Island [1,2,20,55]. At their optimal doses, C15:0 (17 µM) and rapamycin (9 µM) had 24 shared, significant cell-based activities across 10 (83%) out of 12 cell systems, including anti-inflammatory, antifibrotic, and anticancer activities. Our findings are consistent with previously demonstrated anti-inflammatory, antifibrotic, anticancer, and antimicrobial effects of C15:0 and rapamycin in vitro and in vivo [2,26,27,28,57,58,59]. These common activities between C15:0 and rapamycin, which are reasonably expected to enhance healthspan, may be due in part to a shared mTOR-inhibiting mechanism that is a key target for enhancing longevity [23,56].

In addition to rapamycin, the current study identified clinically relevant and cell-based activities shared between C15:0 and metformin. At their optimal doses, C15:0 (17 µM) and metformin (5000 µM) had 11 common, significant cell-based activities across 5 (42%) out of 12 cell systems, including anti-inflammatory activities. These common activities may be due to their shared roles as AMPK activators [22,63]. Consistent with metformin as a treatment for type 2 diabetes, pure C15:0 has been shown to improve glucose uptake by cells, and supplementation over 12 weeks effectively lowers glucose, cholesterol, MCP-1, and IL-6 in a high-fat diet-induced mouse model of type 2 diabetes [2,22]. Further, numerous prospective cohort studies have consistently shown that people with higher circulating C15:0 concentrations have a lower risk of having or developing type 2 diabetes, gestational diabetes, and diabetic retinopathy [3,4,5,6,76,77]. As such, there is a need for clinical trials to evaluate C15:0 as an essential fatty acid that, similar to metformin, may help prevent and attenuate type 2 diabetes.

In our study, acarbose had only five clinically relevant, dose-dependent cell-based activities in the BioMAP system. This outcome is not surprising, given that acarbose exerts its benefits as an α-glucosidase inhibitor that slows the breakdown and digestion of carbohydrates in the gut [35]. Our study supports the primary beneficial role of acarbose at the gut absorption level versus providing systemic cell-based activities. Of interest, eicosapentaenoic acid (EPA), an omega-3 fatty acid that is not a leading longevity-enhancing compound, previously showed only seven dose-dependent cell-based activities in the BioMAP system [25]. While EPA and other omega-3 fatty acids have long been thought to have numerous health benefits, many of these claims have fallen in recent years, especially related to omega-3 marine oil supplements [78,79,80,81,82]. Examples of molecules with lower hits in the BioMAP system, such as acarbose and EPA, help to increase confidence that BioMAP can effectively differentiate molecules based on objectively measured biomarkers at the cellular level.

Importantly, in alignment with our current study showing that C15:0 has cell-based activities matching those of leading longevity-enhancing candidates, there is substantial existing literature supporting C15:0 as a healthspan and longevity-enhancing compound, including expected associations with and direct effects of C15:0 on: (1) hallmarks of aging, (2) aging rate biomarkers, (3) aging-related clinical indices, (4) aging-related diseases, and (5) longevity.

First, as anticipated for a healthspan and longevity-enhancing compound, C15:0 directly targets multiple hallmarks of aging, including mitochondrial dysfunction, cellular senescence, impaired cellular signaling, and inflammaging. Pure C15:0 rescues mitochondrial function at complex II of the mitochondrial respiratory pathway via increased production of succinate and has a dose–response effect on repairing mitochondrial function [2,83,84]. Consistent with the cell membrane pacemaker theory of aging, C15:0, as a stable, odd-chain saturated fatty acid that is readily incorporated in cell membranes, stems premature cellular senescence and lowers the risk of lipid peroxidation [23,35,36,37,38,85,86]. Beyond the role of C15:0 as an mTOR inhibitor and AMPK activator, C15:0 supports healthy cellular signaling as a dual partial PPAR α/δ agonist, JAK-STAT inhibitor, and HDAC6 inhibitor, which are well-established moderators of metabolism, lipids, inflammation, and cancer [2,22,24,26,87,88,89]. As shown in the current study, C15:0 has broad anti-inflammatory activities expected to directly address inflammaging, a chronic, low-level inflammatory state that contributes to the onset and exacerbation of many aging-associated diseases [90].

Beyond targeting multiple hallmarks of aging, C15:0 has been shown to slow the rate at which humans age. Declining hemoglobin, a marker of red blood cell (RBC) loss, has also been identified as a biomarker of an accelerated aging rate [91,92]. This may be due, in part, to the ease of detecting increased fragility of RBCs with age, which serves as a clinically relevant marker of overall cellular fragility with aging [93,94]. Increasing dietary C15:0 effectively raises RBC membrane C15:0 concentrations, which independently predicts raised, healthier hemoglobin concentrations in long-lived mammals [38]. Further, daily supplementation of pure C15:0 (FA15) for 12 weeks successfully increases low hemoglobin in vivo [2]. These studies demonstrate that C15:0 can effectively slow a key biomarker of the aging rate.

In addition to slowing the aging rate, and as would be expected of a healthspan and longevity-enhancing nutrient, C15:0 can positively affect routine clinical indices. Specifically, higher circulating C15:0 concentrations are associated with, and show in relevant animal and cellular models to cause lower inflammation, lower total cholesterol and triglycerides, lower glucose and improved insulin sensitivity, lower liver enzymes, healthier body weight, and, as mentioned above, higher and healthier hemoglobin levels [2,22,24,27,29,30,31,32,33]. Consistent with the broad anti-inflammatory effects of C15:0 shown in our current study, daily oral C15:0 supplementation over 12 weeks lowers the proinflammatory cytokines MCP-1, IL-6, and TNFα, as well as IgG, in animal models of metabolic and liver disease [2]. These effects are aligned with inverse associations found between circulating inflammatory markers (i.e., C-reactive protein and adipokines) and C15:0 in humans [30,31,95]. On the lipid front, daily oral C15:0 supplementation for 12 weeks lowers total cholesterol and triglycerides in animal models of metabolic syndrome and nonalcoholic steatohepatitis [2]. These studies are consistent with inversely correlated circulating C15:0 with total cholesterol, triglycerides, ApoA1, and ApoB in humans [29]. Further, there is limited evidence that C15:0 may support healthier body weight; daily oral C15:0 supplementation lowers body weight gained in high-fat diet-induced obese mice, and circulating C15:0 is inversely correlated with body weight, waist circumference, hip circumference, and BMI in humans [2,32,33]. These studies support the notion that C15:0 has tangible benefits for routinely monitored aging-related clinical indices.

While improving clinical indices is important, a longevity-enhancing compound should effectively delay the onset and progression of chronic diseases that cause mortality: Type 2 diabetes, cancer, and heart disease. In addition to the discussion on type 2 diabetes above, numerous large studies have shown that C15:0 is associated with a lower risk of heart disease, including heart failure, first myocardial infarctions, and coronary heart disease [3,4,5,6,7,8,9,10,11,16,17,18,19]. Further, C15:0 has a strong dose-dependent antiproliferative effect on multiple cancer cell types, including human breast cancer (MCF-7, MDA-MB-231), lung cancer (A549), pancreatic cancer (PANC-1), and liver cancer (HepG2) cells, which have been attributed to JAK2/STAT3 and HDAC6-inhibiting C15:0 activities [24,26]. C15:0 has also been shown to reverse tamoxifen resistance in MCF-7/SC breast cancer cells and improve the efficacy of gemcitabine across multiple cancer cell lines and in vivo [23,28]. Consistent with these studies, the cell-based phenotypic profile of C15:0 at higher doses (50 µM) closely mimics leading and broad anticancer therapeutics, gemcitabine and paclitaxel, which aligns with associations found between higher circulating C15:0 concentrations and lower risks of having breast cancer, small lung cancer, squamous cell carcinoma, colorectal cancer, pharyngolaryngeal cancer, and hematologic malignancies in humans [16,17,18,19,25,96,97]. While these studies are early, they support pure C15:0 as a promising lead or adjunct to safely address multiple types of cancer, a leading cause of mortality.

In addition to type 2 diabetes, heart disease, and cancer, there has been an alarming rise in people with and dying from nonalcoholic fatty liver disease (NAFLD), a metabolic liver disease now affecting 1 out of 3 people, and 1 out of 10 children, globally [98]. Plasma C15:0 concentrations are inversely correlated with liver fat in children, and higher circulating C15:0 is associated with lower ALT, AST, GGT, and less severe NASH in adults [13,15,29,99]. As a dual partial PPAR α/δ agonist, C15:0 has a targeted mechanism of action proposed to treat NAFLD and NASH, and as shown in our current study, C15:0 has direct, dose-dependent, and clinically relevant beneficial activities across multiple primary human cell systems relevant to NASH, including anti-inflammatory and antifibrotic activities; similar benefits have been reported in hepatic cells [2,27,87]. In animal studies, daily oral C15:0 for 11 weeks effectively lowers cholesterol, triglycerides, anemia, inflammation, liver iron deposition, and liver fibrosis in an in vivo model for NAFLD and NASH, and C15:0 has been attributed as the active inulin metabolite, made by the microbiota, responsible for suppressing NASH [2,14]. The precipitous rise in NAFLD globally and the lack of treatments warrant a closer look at C15:0 as a nutrient that may be essential to prevent or delay the onset and progression of NAFLD.

Beyond evidence that C15:0 lowers the risk of conditions that are leading causes of mortality, higher C15:0 has been linked to lower risks of a number of other aging-associated conditions, including anemia, chronic obstructive pulmonary disease, hair loss, and Alzheimer’s disease. Specifically, children with higher erythrocyte cell membrane C15:0 levels have less severe iron deficiency anemia [37], and daily oral C15:0 supplementation successfully attenuates anemia in vivo [2]. Regarding lung disease, dietary C15:0 intake is linearly correlated with improved lung function (FEV1/FVC) in people with COPD [100]. A double-blinded clinical trial demonstrated the efficacy of topical pentadecanoic acid glyceride in treating male pattern alopecia [82,101]. Further, higher free fatty acid C15:0 in cerebrospinal fluid is associated with a lower risk of Alzheimer’s disease [102]. These studies support C15:0 as a healthspan and longevity-enhancing nutrient that can delay the onset and progression of multiple chronic age-related diseases.

While not part of a typical checklist for a healthspan and lifespan-enhancing compound, studies have demonstrated that C15:0 has both antimicrobial and antidepressant properties. Specifically, C15:0 hinders the growth of both bacteria (*Staphylococcus epidermidis* and *Klebsiella pneumoniae*) and fungi (*Candida albicans*) [39,40]. These antimicrobial effects are further supported by C15:0′s cell-based phenotypic profile at moderate concentrations, which closely matches that of clarithromycin and climbazole [25]. Interestingly, both C15:0 and rapamycin have antifungal properties [103].

Science has recently emerged regarding potential C15:0 antidepressant activities. Human cell phenotypic profiling of C15:0 at low to moderate concentrations closely matches those of bupropion, a leading antidepressant, and these results predict that C15:0 may have antidepressant activities [25]. C15:0 antidepressant activities are further supported by the discovery of a key C15:0 metabolite, pentadecanoylcarnitine, which is a full-acting CB1 and CB2 endocannabinoid and serotonin mimic [104]. Consistent with C15:0 having antidepressant-mimicking activities, a study by Brydges et al. showed that people with higher circulating C15:0 concentrations indicated lower anxious-distress dimensions compared to people with lower C15:0 [105]. Interestingly, a large-scale drug screen exploring the effect of many compounds on lifespan in *C. elegans* found that target receptors for biogenic amines, including widely used antidepressants, positively impacted lifespan [106]. Further studies, including demonstrated antidepressant activities in animal models of depression and/or clinical trials, are needed to confirm C15:0 as an antidepressant.

Finally, limited studies have linked higher dietary or circulating C15:0 to longer lifespans. In a prospective cohort study including over 14,000 people followed for an average of 13 years, people with higher dietary intake of odd-chain saturated fatty acids (including C15:0) have a lower risk of mortality in both men and women [107]. While higher circulating C15:0 concentrations are associated with a lower risk of death, not all studies have been consistent [11]. Further, people living in an area in Italy known to have exceptional longevity (sometimes referred to as “Blue Zones” or high longevity zones) have higher circulating C15:0 compared to people living in areas with lower overall longevity [108]. These large epidemiological studies with C15:0, while few, arguably offer a stronger depth of evidence for supporting longevity in humans than rapamycin, metformin, and acarbose, which are prescription drugs intentionally provided to a limited subset of populations to treat disease, thus disabling large population-based studies including healthy individuals.

We recognize some of the limitations of our studies. While dose–response effects were present with C15:0 in the BioMAP panel, there were decreased activities at the highest dose (50 µM). While this could suggest that there were inconsistencies with the assay, this U-shaped activity profile of C15:0 is consistent with a true optimal active concentration of C15:0 at 17 to 20 µM. For example, when mitochondrial repair activities of C15:0 were assessed at 10, 20, 50, 100, and 200 µM, the optimal concentration for lowering mitochondrial reactive oxygen species production was 20 µM [2]. Similarly, while Trieu et al. showed a linear association between higher circulating C15:0 concentrations and lower risk of incident cardiovascular disease, there was a U-shaped association between circulating C15:0 concentration and risk of all-cause mortality; in this study, the lowest risk of mortality was among people with circulating C15:0 concentrations of 0.2 to 0.3% total fatty acids [11]. When including our current findings, these studies support the idea that C15:0 concentrations of 20 µM may be ideal for supporting human longevity. It is important to note, however, that our definition of “optimal dose” for all four compounds was based on concentrations that resulted in the most BioMAP biomarker hits, which may or may not translate into optimal doses proven to enhance longevity in vivo.

Although we used an independent lab to conduct a state-of-field industry standard panel of 148 assays (i.e., we contracted with the Eurofins lab and did not run the assays using our own facilities), the BioMAP Diversity PLUS system may not capture everything of relevance to true healthspan and longevity-enhancing interventions. Also, since the activity profiles of the compounds tested did not impact all the assays tested, it could be that certain processes of relevance to true healthspan and longevity-enhancing interventions were not affected by the compounds. In addition, the assays are cell based, which of course comes with caveats about the translation of our findings to human in vivo biology. Finally, it could be that, as many believe, true healthspan and longevity-enhancing interventions should simultaneously slow the aging rate and not just impact systems implicated in disease processes, say, secondarily [44,45,46,47]. Just how one could measure the aging rate in various drug screening systems is unclear, as is the direct connection between the aging rate, however defined, and age-related disease processes [109], although this is an important and growing area of research [44,45,46,47]. However, we believe the disease models we explored, given that they touch on a wide variety of disease processes known to impact, or be impacted by, aging are useful screening tools for any and all candidate healthspan and longevity-enhancing interventions. Further, our extensive review of the literature provided ample evidence that the presented cell-based screening results closely align with C15:0 as a valid candidate healthspan and longevity-enhancing intervention. We have argued elsewhere that it would be a hard argument to make that a true healthspan and longevity-enhancing intervention works through mechanisms so cryptic that they in fact do not impact any of the known and validated disease processes explored in modern pharmaceutical research [109]. In this light, compounds with more activities in the BioMAP Diversity PLUS panel than those studied here that also have no adverse effects should also be considered as candidate healthspan and longevity-enhancing interventions.

There is an added urgency to further evaluate C15:0 as a healthy aging nutrient. Due in part to population-wide decreased intake of whole-fat dairy products (our primary dietary source of C15:0), circulating C15:0 concentrations have been declining while health issues have risen, suggesting a possible ecological link between decreased C15:0 and increased disease [2,110]. Further, circulating C15:0 concentrations decrease with advancing age, suggesting a possible link with age-related diseases [38,111]. With these facts in mind, it has been suggested that global nutritional C15:0 deficiencies may be contributing to the rise in chronic age-related conditions [2,25].

## 5. Conclusions

In summary, our studies and prior literature show that C15:0 and rapamycin, a leading healthspan and longevity-enhancing intervention candidate, share numerous clinically relevant activities, including anti-inflammatory, anticancer, antifibrotic, antimicrobial, and mTOR-inhibiting activities. Further, as an AMPK activator, C15:0 also showed multiple similarities to metformin, especially at specific doses. Above and beyond in vitro and in vivo studies, C15:0 has the added benefit over rapamycin and metformin of having its associations with health studied in global meta-analyses of large prospective cohort studies that have taken place over decades, most of which included healthy individuals; these studies consistently show that people with higher circulating C15:0 concentrations have a lower risk of aging-associated conditions. Given the voluminous supporting literature, we propose C15:0 as a natural, effective, and safe odd-chain saturated fatty acid with strong evidence that this essential nutrient supports healthy aging and longevity in humans, with cell-based activities that are as good as, or better than, leading longevity-enhancing prescription therapeutics. Given population wide declining C15:0 levels, there is a need to evaluate the potential effects of nutritional C15:0 deficiencies on our healthspan and longevity.

## 6. Patents

Patents relevant to this manuscript are available at DiscoverC15.com/patents (accessed on 25 October 2023).

## Figures and Tables

**Table 1:** Dose–response annotated activities of C15:0 (FA15), rapamycin, metformin, acarbose, and omega-3 (eicosapentaenoic acid, EPA, as previously published [25]) among 148 biomarkers and 12 primary human cell systems mimicking various disease states. Biomarker activities were annotated when 2 or more consecutive concentrations changed in the same direction relative to vehicle controls, were outside of the significance envelope and had at least one concentration with an effect size > 20% (|log10 ratio| > 0.1).

BioMAP Phenotypic Cell Profiles	Dose-Dependent Activities on Clinically Relevant Biomarkers				
	C15:0 (FA15)	Rapamycin	Metformin	Acarbose	Omega-3 (EPA) [25]
Dose ranges	1.9–50 µM	0.3–9 µM	190–5000 µM	1.1–30 µM	1.9–17 µM
Dietary supplement (DS) or prescription drug (Rx) ingredient	DS	Rx	Rx	Rx	DS
Total number (%) of systems with dose-dependent activities	10 (83%)	12 (100%)	7 (58%)	3 (25%)	4 (33%)
Total number of biomarkers with dose-dependent changes	36	32	17	5	7

**Table 2:** Dose-dependent annotated activities on clinically relevant biomarkers of: C15:0 (FA15) (1.9, 5.6, 17, 50 µM), rapamycin (0.3, 1, 3, 9 µM), metformin (190, 560, 1700, 5000 µM), and acarbose (1.1, 3.3, 10, 30 µM) among 12 primary human cell systems mimicking various disease states. Eicosapentaenoic acid (EPA), previously published, is included as a reference [25]. Based on the accepted BioMAP procedures, biomarker activities were annotated when 2 or more consecutive concentrations changed in the same direction relative to vehicle controls, were outside of the significance envelope, and had at least one concentration with an effect size >20% (|log10 ratio| > 0.1). Up arrows indicate that the measured biomarker was significantly higher than the significance envelope (higher than non-treated vehicle controls); down arrows indicate that the measured biomarker was significantly lower than the significance envelope (lower than non-treated vehicle controls).

BioMAP Phenotypic Cell Profiles			Dose-Dependent Annotated Activities by Compounds on Clinically Relevant Biomarkers				
BioMAP Cell System	Human Cell Types and Stimulation	Disease Relevance	C15:0 (FA15^TM^) (1.9–50 µM)	Rapamycin (0.3–9 µM)	Metformin (190–5000 µM)	Acarbose (1.1–30 µM)	Omega-3 (EPA) [25] (1.9–17 µM)
3C	Venular endothelial cells stimulated with TNFα, IL-1β, IFNγ	Cardiovascular disease, chronic inflammation	↓ HLA-DR ↓ MCP-1 ↓ proliferation	↓ HLA-DR ↓ VCAM-1 ↓ uPAR ↓ proliferation	↓ HLA-DR ↓ IL-8	None	↓ MCP-1 ↓ uPAR
4H	Venular endothelial cells stimulated with IL-4, histamine	Autoimmunity, allergy, asthma	↓ Eot3	↓ MCP-1	None	None	None
LPS	Venular endothelial cells and peripheral blood mononuclear cells stimulated with TLR4 ligand	Chronic inflammation, cardiovascular disease	↓ VEGFR2 ↓ MCP-1 ↓ CD69 ↓ IL-1α	↓ CD40	↓ CD40	None	None
SAg	Venular endothelial cells and peripheral blood mononuclear cells stimulated with TCR ligands	Chronic inflammation, autoimmune disease	↓ CD38 ↓ CD40 ↓ CD69 ↓ T cell proliferation	↓ CD38 ↓ CD40 ↓ MCP-1 ↓ T cell proliferation	↓ CD38 ↓ CD69 ↓ T cell proliferation	None	None
BT	Peripheral blood mononuclear cells and B cells stimulated with αIgM, TCR ligands	Asthma, cancer, autoimmunity, allergy	↓ IgG ↓ IL-17F	↓ IgG ↓ IL-17F ↓ TNFα ↓ IL-6 ↓ IL-2 ↓ IL-17A ↓ proliferation	None	None	None
BF4T	Bronchial epithelial cells and dermal fibroblasts stimulated with IL-4, TNFα	Fibrosis, lung inflammation, asthma, allergy	None	↓ VCAM-1 ↓ tPA	↓ MCP-1 ↓ tPA	None	↓ PAI-I
BE3C	Bronchial epithelial cells stimulated with IL-1β, IFNγ, TNFα	COPD, lung inflammation	↓ tPA	↓ tPA	↓ IP-10 ↓ IL-8 ↓ HLA-DR ↓ MMP9	None	None
CASM3C	Coronary artery smooth muscle cells stimulated with IL-1β, TNFα, IFNγ	Cardiovascular inflammation, restenosis	↓ HLA-DR ↓ IL-6 ↓ VCAM-1 ↓ TM ↓ TF ↓ proliferation	↓ HLA-DR ↓ uPAR ↓ proliferation	None	↓ uPAR	None
HDF3CGF	Dermal fibroblasts stimulated with IFNγ, TNFα, IL-1β, EGT, bFGF, PDGF-BB	Fibrosis, chronic inflammation	↓ PAI-1 ↓ VCAM-1 ↓ IP-10 ↓ ITAC ↓ MIG ↓ fibroblast proliferation	↓ PAI-I ↓ EGFR ↓ fibroblast proliferation	↓ Col-III	↓ EGFR ↓ fibroblast proliferation	↓ PAI-I ↓ M-CSF
KF3CT	Keratinocytes, dermal fibroblasts stimulated with IL-1β, IFNγ, TGFβ, TNFα	Dermatitis, psoriasis	None	↓ PAI-I	None	None	None
MyoF	Lung fibroblasts stimulated with TGFβ, TNFα	Wound healing, matrix remodeling, fibrosis, chronic inflammation	↓ VCAM-1 ↓ Col-I ↓ IL-8 ↓ Decorin ↓ TIMP-1	↓ VCAM-1 ↓ PAI-I	None	↓ Col-IV ↓TIMP1	↓ Col-I ↓ Col-III
/Mphg	Macrophages and venular endothelial cells stimulated with TLR2 ligand	Chronic inflammation, restenosis, cardiovascular disease	↓ CD40 ↓ CD69	↓ sIL-10	↓ MCP-1 ↓ Esel ↓ IL-8	None	None

**Table 3:** Number of biomarker hits by compound and dose, of C15:0 (FA15) (D1 = 1.9, D2 = 5.6, D3 = 17, D4 = 50 µM), rapamycin (D1 = 0.3, D2 = 1, D3 = 3, D4 = 9 µM), metformin (D1 = 190, D2 = 560, D3 = 1700, D4 = 5000 µM), and acarbose (D1 = 1.1, D2 = 3.3, D3 = 10, D4 = 30 µM) among 12 primary human cell systems and 148 biomarkers mimicking various disease states. Biomarker hits include clinically relevant biomarker measurements in treated systems that were outside of the significance envelope (i.e., significantly different than non-treated control systems). Optimal dose was defined as the dose with the most biomarker hits.

Compound	Number of Biomarker Hits by Increasing Dose				
	D1 Lowest Dose	D2 Lower Dose	D3 Higher Dose	D4 Highest Dose	Optimal Dose (μM)
C15:0 (FA15)	28	56	81	40	17
Rapamycin	55	62	61	75	9
Metformin	9	5	28	53	5000
Acarbose	15	17	24	28	30

**Table 4:** Clinically relevant activities at optimal doses for C15:0 (FA15,17 µM), rapamycin (9 µM), and metformin (5000 µM) among 12 primary human cell systems mimicking various disease states. Based on the accepted BioMAP procedures, biomarker activities were considered significant when the biomarker measurement was outside of the significance envelope and had an effect size ≥20% (|log10 ratio| ≥ 0.1) compared to non-treated control systems. Up arrows indicate that the measured biomarker was significantly higher than the significance envelope (higher than non-treated vehicle controls); down arrows indicate that the measured biomarker was significantly lower than the significance envelope (lower than non-treated vehicle controls).

BioMAP Cell System	Disease Relevance	Significant Activities on Clinically Relevant Biomarkers at Each Compound’s Optimal Dose		
		C15:0 (FA15)	Rapamycin	Metformin
Optimal Dose		17 µM	9 µM	5000 µM
3C	Cardiovascular disease, chronic inflammation	↓ MCP-1, ↓ HLA-DR, ↓ SRB ↓ endothelial cell proliferation, ↑ thrombomodulin, ↓ IL-8	↓ MCP-1, ↓ HLA-DR, ↓ SRB ↓ endothelial cell proliferation, ↓ VCAM-1, ↓ uPAR	↓ HLA-DR, ↓ uPAR, ↓ IL-8
4H	Autoimmunity, allergy, asthma	↓ eotaxin-3, ↓ VEGFR	↓ MCP-1, ↓ SRB	↓ P-selectin
LPS	Chronic inflammation, cardiovascular disease	↓ MCP-1, ↓ VCAM-1, ↑ tissue factor, ↓ CD40, ↓ SRB ↓ CD69, ↑ thrombomodulin, ↓ IL-1α	↓ MCP-1, ↓ VCAM-1, ↑ tissue factor, ↓ CD40, ↓ SRB ↓ M-CSF, ↑ PGE2,	↓ MCP-1, ↓ CD40, ↓ CD69, ↑ IL-1α, ↑ PGE2, ↑ TNFα
SAg	Chronic inflammation, autoimmune disease	↓ CD38, ↓ CD40, ↓ CD69, ↓ T cell proliferation, ↓ SRB	↓ CD38, ↓ CD40, ↓ T cell proliferation, ↓ SRB, ↓ MCP-1	↓ CD38, ↓ CD40, ↓ T cell proliferation, ↓ CD69, ↓ IL-8
BT	Asthma, cancer, autoimmunity, allergy	↓ sIgG, ↓ sIL-17A, ↓ sIL-17F, ↓ TNFα	↓ sIgG, ↓ sIL-17A, ↓ sIL-17F, ↓ TNFα, ↓ B cell proliferation, ↓ sIL-2, ↓ sIL-6	↓ sIgG, ↓ TNFα, ↓ sIL-6
BF4T	Fibrosis, lung inflammation, asthma, allergy	None	↓ tPA, ↓ VCAM-1	↓ tPA, ↓ MCP-1, ↓ eotaxin-3, ↓ IL-8, ↓ MMP-3, ↓ MMP-9
BE3C	COPD, lung inflammation	↓ PAI-I, ↓ tPA	↓ PAI-I, ↑ MMP-1	↓ tPA, ↓ IL-8, ↓ HLA-DR, ↓ MMP-9
CASM3C	Cardiovascular inflammation, restenosis	↓ HLA-DR, ↓ VCAM-1, ↓ thrombomodulin, ↓ tissue factor	↓ HLA-DR, ↓ uPAR, ↓ coronary artery proliferation	None
HDF3CGF	Fibrosis, chronic inflammation	↓ PAI-I, ↓ fibroblast proliferation, ↓ MCP-1, ↓ VCAM-1, ↓ IP-10, ↓ I-TAC, ↓ MIG	↓ PAI-I, ↓ fibroblast proliferation, ↓ EGFR	↓ VCAM-1, ↓ collagen-III
KF3CT	Dermatitis, psoriasis	↓ PAI-I	↓ PAI-I	None
MyoF	Wound healing, matrix remodeling, fibrosis, chronic inflammation	↓ VCAM-1, ↓ collagen-I, ↓ collagen-III, ↑ collagen-IV, ↓ decorin, ↓ TIMP-1	↓ VCAM-1, ↑ IL-8	None
/Mphg	Chronic inflammation, restenosis, cardiovascular disease	↓ sIL-10, ↓ CD40, ↑ MIP-1α, ↑ E-selectin, ↓ CD69	↓ sIL-10, ↓ E-selectin	↓ E-selectin, ↓ MCP-1, ↓ IL-8

## Data Availability

All data generated or analyzed during this study are included in this published article and its supplementary information files.

## Supplementary Materials

The following supporting information can be downloaded at: https://www.mdpi.com/article/10.3390/nu15214607/s1. Raw data from BioMAP Diversity Plus are provided as Supplementary Materials. Table S1. BioMAP^®^ Diversity PLUS^®^ measured changes across 12 primary human cell systems with 148 clinically relevant biomarkers compared to non-treated control systems for acarbose, metformin, rapamycin and C15:0 (FA15) at four doses. Table S2. BioMAP^®^ Diversity PLUS^®^ cytotoxicity data for acarbose, metformin, rapamycin and C15:0 (FA15) at four doses. Table S3. BioMAP^®^ Diversity PLUS^®^ number of biomarker hits, by dose, for acarbose, metformin, rapamycin and C15:0 (FA15) at four doses.

## Abbreviations

ALT	alanine transaminase
AMPK	5′ adenosine monophosphate-activated protein kinase
ApoA1	apolipoprotein A1
ApoB	apolipoprotein B
AST	aspartate aminotransferase
bFGF	basic fibroblast growth factor
C15	0:pentadecanoic acid
CB1	cannabinoid 1 receptor
CB2	cannabinoid 2 receptor
Coll-I	collagen I
Coll-III	collagen III
Coll-IV	collagen IV
COPD	chronic obstructive pulmonary disease
EGF	epidermal growth factor
EGFR	epidermal growth factor receptor
Eot3	eoxtaxin-3
EPA	eicosapentaenoic acid
Esel	E-selectin
FEV1/FVC	forced expiratory volume in 1 s/forced vital capacity
GGT	gamma-glutamyl transferase
HDAC-6	histone deacetylase 6
HDFn	human neonatal dermal fibroblasts
HLA-DR	major histocompatibility complex II cell surface receptor
HUVEC	human umbilical vein endothelial cells
IFNɣ	interferon gamma
IL-1α	interleukin 1α
IL-1β	interleukin 1β
IL-4	interleukin 4
IL-6	interleukin 6
IL-8	interleukin 8
IL-10	interleukin 10
IL-17A	interleukin 17A
IL-17F	interleukin 17F
IP-10	interferon-gamma inducible protein of 10 kDa
ITAC	interferon-inducible T-cell alpha chemoattractant
JAK-STAT	Janus kinase-signal transducers and activators of transcription
LPS	lipopolysaccharide
MCP-1	monocyte chemoattractant protein-1
M-CSF	macrophage colony-stimulating factor
MIG	monocyte induced by gamma interferon
MIP-1α	macrophage inflammatory protein-1 alpha
MMP3	matrix metalloproteinase-9
MMP9	matrix metalloproteinase-9
mTOR	mechanistic target of rapamycin
NAFLD	nonalcoholic fatty liver disease
NASH	nonalcoholic steatohepatitis
PAI-I	plasminogen activator inhibitor-1
PBMC	primary blood mononuclear cells
PDGF-BB	platelet derived growth factor subunit B
PGE2	prostaglandin E2
PPAR-α/δ	peroxisome proliferator-activated receptors alpha/delta
RBC	red blood cell
SRB	sulforhodamine B
TCR	T-cell receptor
TF	tissue factor
TGFβ	transforming growth factor-β
TIMP-1	tissue inhibitor of metalloproteinase-1
TM	thrombomodulin
TLR2	toll-like receptor 2
TLR4	toll-like receptor 4
TNFα	tumor necrosis factor alpha
tPA	tissue plasminogen activator
uPAR	urokinase receptor
VCAM-1	vascular cell adhesion molecule 1
VEGFR2	vascular endothelial growth factor receptor 2
